# MicroRNAs as Biomarkers for Ionizing Radiation Injury

**DOI:** 10.3389/fcell.2022.861451

**Published:** 2022-03-03

**Authors:** Meng Jia, Zhidong Wang

**Affiliations:** Department of Radiobiology, Beijing Key Laboratory for Radiobiology, Beijing Institute of Radiation Medicine, Beijing, China

**Keywords:** microRNA, ionizing radiation, injury assessment, biomarker, body fluids

## Abstract

Accidental radiation exposures such as industrial accidents and nuclear catastrophes pose a threat to human health, and the potential or substantial injury caused by ionizing radiation (IR) from medical treatment that cannot be ignored. Although the mechanisms of IR-induced damage to various organs have been gradually investigated, medical treatment of irradiated individuals is still based on clinical symptoms. Hence, minimally invasive biomarkers that can predict radiation damage are urgently needed for appropriate medical management after radiation exposure. In the field of radiation biomarker, finding molecular biomarkers to assess different levels of radiation damage is an important direction. In recent years, microRNAs have been widely reported as several diseases’ biomarkers, such as cancer and cardiovascular diseases, and microRNAs are also of interest to the ionizing radiation field as radiation response molecules, thus researchers are turning attention to the potential of microRNAs as biomarkers in tumor radiation response and the radiation toxicity prediction of normal tissues. In this review, we summarize the distribution of microRNAs, the progress on research of microRNAs as markers of IR, and make a hypothesis about the origin and destination of microRNAs *in vivo* after IR.

## Introduction

The threat of accidental exposure to ionizing radiation is growing. Victims exposed to more than 1 Gy of IR can develop acute radiation syndrome (ARS) (Christensen et al., 2014), which affects the hematopoietic and gastrointestinal systems and causes radiation injury. The progression of ARS in humans exposed to high doses of radiation can be very rapid, which poses a great challenge for subsequent medical treatment. Therefore, the identification of radiation injury is extremely crucial. Chromosomal aberration analysis, lymphocyte γ-H2AX foci analysis and lymphocyte count analysis are all existing biological dose estimation techniques (Wang et al., 2016). Despite the high sensitivity and specificity of those techniques, they still have limitations such as the complicated detection process, high technical difficulty, and the need for multiple measurements. Thus, research on molecular biomarkers of ionizing radiation injury is needed to achieve early and rapid diagnosis of casualties.

MicroRNAs (miRNAs) are small non-coding RNA molecules of 19–25 nucleotides in length, which play a role in gene regulation (Bartel, 2004) and are promising biomarkers that have emerged in recent years under different pathological conditions. The pathogenesis of various diseases ranging from cancer to autoimmune and cardiovascular diseases involves miRNAs regulation (Mendell and Olson, 2012). miRNAs are detected in body fluids, such as serum and plasma, and several studies have explored the potential of serum or plasma miRNAs as biomarkers for diseases such as cancer and cardiovascular diseases (Cortez and Calin, 2009; Cortez et al., 2011). In addition to those pathological conditions, several studies have investigated the association between IR and miRNAs, and researchers have found that many miRNAs are dysregulated in expression under IR (Czochor and Glazer, 2014). Subsequently, it was found that miRNAs played an important role in the radiation response of cells by regulating the corresponding target genes involved in DNA damage repair, cell cycle checkpoint and apoptosis (Zhao et al., 2013). Hence, the possibility of miRNA as a biomarker of IR is being explored.

## The Distribution of miRNAs

MicroRNA is a short, non-coding RNA that acts as a post-transcriptional regulator of gene expression. The process of miRNAs production has been elucidated (Bartel, 2004). Over 2500 mature miRNA sequences have been identified in the human body (Kozomara et al., 2019), and miRNAs are predicted to control the activity of around 50% of all protein-coding genes in mammals (Krol et al., 2010). Intracellular miRNAs are involved in many biological activities, such as stem cell differentiation, organ development, cell death, cell cycle phase transition and signal transduction, however, the functions played by extracellular free miRNAs are not yet clear.

### miRNAs in Organs and Tissues

Explaining clearly miRNAs tissue or organ enrichment and specificity helps evaluate its ability to act as biomarkers of tissue damage caused by stress, diseases or toxicants. Some research reported the specific and enriched sites of miRNAs analyzed through the number of miRNAs present in various tissues and organs of rats and humans, which were detected via high-throughput sequencing. Bushel et al. (Bushel et al., 2020) constructed a tissue atlas of miRNA abundance with miRNAs sequencing data of tissues in rats. The atlas contains three bioinformatics pipelines to detect abundant miRNAs. One pipeline analysis showed that 231 tissue-enriched miRNAs were found in the spinal ganglia, followed by 191 miRNAs in whole blood, 186 miRNAs in the brainstem and 92 miRNAs in the stomach. It is noteworthy that the number of miRNAs enriched in some tissues such as whole blood, varied considerably between pipelines (pipeline 1: 25; pipeline 2: 191). Different bioinformatics pipelines yielded different results, probably because there is no standard method for analyzing RNA sequencing data, so miRNAs detected by multiple methods are more likely to be tissue-enriched and tissue-specific. Panwar et al. (Panwar et al., 2017) used 304 high-quality microRNA sequencing datasets from NCBI-SRA to calculate the expression profiles for different human tissues and cell lines. The study summarized the five most highly expressed miRNAs in each tissue and cell line, such as let-7f-5p, let-7b-5p, let-7a-5p, miR-140-3p and miR-423-5p in lung, miR-143-3p, miR-10a-5p, miR-122-5p, miR-22-3p and miR-192-5p in liver. For tissue-specific miRNAs, Landgraf et al. (Landgraf et al., 2007) provided some evidence. They sequenced 256 small RNA libraries from a total of 26 different organ systems and cell types in humans and rodents, and their analysis showed that some highly conserved miRNAs in humans and rodents had the same tissue-specific expression, such as miR-302a in the embryo, miR-122 in the liver, miR-9 and miR-124 in the nervous system. Afterwards, Ludwig et al. (Ludwig et al., 2016) presented a tissue atlas of human miRNAs, using a total of 61 (24 and 37 samples collected respectively) tissue biopsies from different organs of two patients to analyze the abundance of the then known 1997 mature miRNAs. Excluding the 633 undetected miRNAs, 1364 miRNAs’ expression was detected in the collected tissues, of which 143 miRNAs were broadly expressed, such as has-miR-1246, has-miR-718, hsa-miR-21-5p, hsa-miR-150-3p and hsa-let-7b-5p. The team also validated the Landgraf data, confirming the specific expression of miR-122 in the liver, miR-9 and miR-124 in the brain and miR-7 in the pituitary. Regarding whether various members of the miRNA family are similarly expressed in specific organs, they found that most members of the miRNA-506 family showed high abundance in the testis and less expression in other tissues. However, the five members of the miR-449 family did not share the same expression pattern: miR-449c-3p and miR-449b-3p were specifically expressed in spleen tissue, while miR-449c-5p and miR-449b-5p were specifically expressed in the kidney and small intestine, and miR-449a was specifically expressed in the lung, kidney and brain, suggesting that miRNAs from the same family may function in different parts of the body.

### Extracellular miRNAs

Many studies have reported that miRNA is a non-invasive or minimally invasive biomarker, existed in not only solid tissues but in various body fluids [including serum (Duan et al., 2021), urine (Hanke et al., 2010), saliva (Park et al., 2009), milk (Gu et al., 2014) etc.]. Furthermore, the amount and type of extracellular miRNAs are significantly different in different types of body fluids (Weber et al., 2010). Weber et al. (Weber et al., 2010) detected the number of miRNAs in 12 types of body fluids. In that study, the largest numbers of miRNAs detected in saliva was 458 miRNAs, followed by semen, breast milk, and peritoneal fluid. The minimum number of miRNAs measured in urine was 204 miRNAs and 61 miRNAs were detected in all body fluid types. Some high-abundance miRNAs (e.g., miR-509-5p, miR-515-3p and miR-335) were distributed in different types of body fluids, suggesting that these miRNAs may have a common function or origin. Based on the currently detectable miRNA expression profiles, several miRNAs were present only in specific types of body fluid, such as miR-224 in plasma, miR-637 in tear fluid, miR-193b in breast milk, and miR-508-5p in semen. Additionally, plasma-specific miRNAs were the most diverse, followed by saliva.

Due to the high amount and investigable value of specific miRNAs in plasma, researchers have tried to figure out the origin of these molecules when exploring their function. The question of whether the miRNAs detected in serum or plasma are released as a result of cell rupture (particularly of blood cells), or whether they are actively secreted by cells has not been answered definitively. People speculated on three sources. First, because of the large number of blood cells present in serum or plasma, the vast majority of miRNAs in serum or plasma is likely to come from blood cells. Studies have shown that circulating cancer biomarker miRNAs are also expressed in blood cells (Pritchard et al., 2012) (e.g., miR-92a, miR-496-5p, miR-16 and miR-451 in erythrocytes), thus blood cell abundance affects the levels of circulating miRNAs. Second, miRNAs in tissues also have a significant effect on extracellular miRNAs. The most obvious evidence is the stable presence of tissue-specific miRNAs in plasma as well, such as liver-specific miR-122 and brain-specific miR-124. Third, tumor cells also actively release miRNAs into the circulatory system. Many tumor-specific miRNAs are detected in the circulatory system at different stages of disease development, and some studies have shown that miRNAs in plasma or serum are expressed as consistently as those in tumors. However, how organs and tissues influence plasma and serum miRNA levels in both pathological and normal states require further experiments to elucidate. When miRNAs in plasma and serum have been extensively studied, researchers have also focused on the identification and function of miRNAs in other body fluids such as saliva, urine and milk.

## Potential of miRNAs as Biomarkers of Exposure to Ionizing Radiation

The increasing number of studies using miRNAs as biomarkers indicated that miRNAs were suitable for diagnosis, prognosis and therapeutic monitoring of various diseases. The most effective biological samples for biomarker detection are urine and plasma (Byrum et al., 2017). Studies on miRNA as a biomarker for various diseases such as cancer made great progress. Although there is little research of miRNA biomarkers in the field of radiation, some discoveries have been made in the study of miRNA as a biomarker for the evaluation of ionizing radiation exposure. The following summarizes some studies of miRNAs in body fluids as biomarkers of exposure to IR.

### Peripheral Blood Cells

Blood is readily available and relatively non-invasive, which is extremely suitable for the discovery and detection of biomarkers. Changes in the number of various cell types in the blood after IR exposure directly reflect the effects of radiation on the body, corresponding to changes in the composition of substances within the blood cells. Several articles have shown that IR induces miRNA specific alterations in whole blood cells. Templin et al. (Templin et al., 2011) found that IR-induced miRNA signals in mouse blood revealed radiation type-specific and dose-dependent expression. The researchers irradiated mice with gamma-ray (representing low LET radiation, groups: 0, 0.5, 1.5 and 5 Gy) and ^56^Fe ions (representing high LET radiation, groups: 0, 0.1 and 0.5 Gy) under total body irradiation (TBI) conditions, and collected blood from the jaws of mice 6 and 24 h after irradiation. They detected 31 miRNAs that were differentially expressed after irradiation with radiation type specificity. There were 11 differentially expressed miRNAs 6 h after γ-irradiation (miR-10a, miR-135a, miR-135b, miR-139-5p, miR-147, miR-200b, miR-223, miR-450a-5p, miR-547, miR-598, miR-708), 6 differentially expressed miRNAs 24 h after γ-irradiation (miR-146a, miR-151-3p, miR-335-3p, miR-337-3p, miR-339-3p, miR-667), and 6 differentially expressed miRNAs by ^56^Fe ions irradiation (miR-350, miR-379, miR-383, miR-409-3p, miR-494, miR-879). miR-150 was present under all three irradiation conditions, miR-511 was differentially expressed at both time points of γ-irradiation, and the remaining miRNAs belonged to the intersection group between the two time points after γ-irradiation and ^56^Fe ions irradiation, respectively (6 h after γ-irradiation and ^56^Fe irradiation: miR-680, miR-685; 24 h after γ-irradiation with ^56^Fe irradiation: miR-125a-3p, miR-211, miR-342-3p, miR-501-3p). In addition to radiation type-specific miRNAs, they found 4 miRNAs with a trend of increased expression levels with increasing dose: miR-135a was elevated 4-fold at 1.5 Gy and 19.9-fold at 5 Gy after 6 h of γ-irradiation compared to the unirradiated group; miR-147 was increased from 80-fold at 1.5 Gy to 215-fold at 5 Gy; miR-680 was increased from 182.8-fold at 1.5 Gy to 2530-fold at 5 Gy; miR-685 was increased from 170-fold at 1.5 Gy to 2430-fold at 5 Gy. According to the data in this article, those miRNAs showing high levels expression after irradiation are suitable to the application of biomarker molecules, and miR-147, miR-680 and miR-685 are only expressed after irradiation, so they may be used to determine whether the subject has been irradiated.

Peripheral Mononuclear Blood Cells (PBMCs), which include lymphocytes and monocytes, are particularly sensitive to IR (Bauer et al., 2011) and used as a surrogate tissue for injury-based radiation biodosimetry (IAEA, 2001). Lee et al. (Lee et al., 2014) irradiated the collected human peripheral blood with 0.5, 1, 2.5 and 5 Gy (^60^Co, dose rate of 0.546 Gy/min) and isolated PBMCs for miRNA microarray analysis 24 h after irradiation. The majority of miRNAs were up-regulated at 1 Gy and below, while only significantly down-regulated miRNAs were found in the 5 Gy irradiated group. Compared to the non-irradiated group, only miR-185-5p was up-regulated significantly in 0.5 Gy group. And 1 Gy group showed 7 up-regulated and 2 down-regulated miRNAs(up-regulated: miR-107, miR-126-3p, miR-144-3p, miR-17-5p, miR-185-5p, miR-20b-5p, miR-5194; down-regulated: miR-3180, miR-4730), 4 miRNAs (miR-142-3p, miR-142-5p, miR-223-3p, miR-451a) with down-regulated expression in the 5 Gy group, whereas no notably expressed miRNA in 2.5 Gy group. Afterwards, the researchers found that differentially expressed miRNAs may regulate some genes that induce apoptosis under IR, and therefore it could explain the IR-induced damage to cells, confirming that tissues and cells damage alter miRNA expression and on the other hand altered miRNA expression reflects changes in cells after IR exposure. The expression of miRNAs in PBMCs after irradiation may vary according to the gravitational state, and astronauts on space missions may be more susceptible to the impacts of radiation due to being in a weightless state. Girardi et al. (Girardi et al., 2012) examined the miRNA expression profiles of human peripheral blood lymphocytes, which were irradiated with γ-rays at 0.2 and 2 Gy and then incubated for 4 or 24 h in normal and simulated microgravity conditions, respectively. The researchers speculated that weightlessness may have a synergistic effect on cells together with IR, affecting the expression of miRNAs involved in DNA damage repair. The researchers conducted an experiment to simulate the cellular response to IR under microgravity conditions (weightlessness) and found that miRNA expression also had a dose effect and a time effect, but was less radiation-responsive compared to miRNA expression under normal gravity conditions. It was evidenced by a lower number of radiation-responsive miRNAs under microgravity conditions than under normal gravity conditions at 4 and 24 h post-irradiation, 0.2 and 2 Gy. At 24 h post-irradiation, miR-34a and miR-34b were both up-regulated 2-3fold under two conditions; miR-144 and miR-598 were significantly down-regulated and miR-27a expression was up-regulated under microgravity conditions. Based on the miRNA expression profiles, we could screen for differentially expressed miRNAs induced by radiation in peripheral blood lymphocytes, which could be used as candidate molecules for radiation biomarkers, while giving hints on the regulation of biological effects of IR in which miRNAs are involved.

Although blood cells are more radiosensitive, when exposed to IR the number of blood cells would be drastically reduced, making it difficult to determine whether the detected decreased differential miRNA signals are due to the altered cell function or the reduction in cell numbers, which could hinder subsequent studies of the specific functions of miRNAs.

### Plasma or Serum

Due to the large variety of blood cells, it is hard to analyze the origin of miRNAs in blood accurately, and thus much research has turned to free miRNAs in plasma or serum. Initially from clinical cancer samples, researchers identified a number of miRNAs in plasma or serum that could serve as markers for radiotherapy. Summerer et al. (Summerer et al., 2013) identified several miRNAs in plasma that could distinguish samples from head and neck squamous cell carcinoma (HNSCC) patients before and after radiotherapy. The samples were plasma of 18 HNSCC patients treated with radiotherapy, who were clinically irradiated with X-rays in fractions (2 Gy per day, 5 days per week, accumulating 70 Gy) at the site of the tumor, and blood was collected before the first irradiation and 1 h after the second irradiation. They found that six miRNAs(miR-425-5p, miR-21-5p, miR-106b-5p, miR-590-5p, miR-574-3p, miR-885-3p) could distinguish plasma from unirradiated and irradiated HNSCC patients by a clustering algorithm. In order to validate the radiation-responsive miRNAs in plasma and further identify associations between those miRNAs and prognostic indicators, the team followed up with a validation cohort study (Summerer et al., 2015), in which plasma miRNAs were assessed in 11 patients with HNSCC who received radiotherapy (radiotherapy as described above). This independent cohort study reconfirmed the previous radiotherapy-responsive plasma miRNAs, identifying that high expression levels of miR-186-5p, miR-374b-5p and miR-574-3p in pre-treatment plasma were associated with shorter progression-free survival or overall survival, and highly expressed miR-28-3p, miR-142-3p, miR-191-5p, miR-195-5p, miR-425-5p and miR-574-3p in post-treatment plasma were associated with poorer prognosis. In addition, high expression of plasma miR-186-5p and miR-374b-5p before treatment was significantly associated with lower local tumor control rates, and high expression of plasma miR-142-3p after treatment was shown to be a marker of decreased local tumor control rates and progression-free survival. Han et al. (Han et al., 2020) found that low expression of miR-26b-5p in serum was related with reduced survival of patients with lung adenocarcinoma after radiotherapy. Tomasik et al. (Tomasik et al., 2021) used serum miRNAs (miR-425-5p and miR-185-5p) to predict the incidence of severe xerostomia after radiotherapy in patients with nasopharyngeal carcinoma. This study is the first to apply molecular test for predicting this severe complication, they found miRNAs carry information that is not directly related to the physical dose delivered and thus they speculated that the miRNA signature reflects the impacts of radiation on the health of the organism rather than the physical dose. Additional studies focused on specific miRNA biomarkers in clinical samples also showed the predictive capability for prognosis after radiotherapy (details in Table 1).

**TABLE 1:** Serum or plasma miRNAs as IR biomarkers in human samples.

miRNAs	Material	Species	Predictors	References
miR-199a-5p↑	Serum	Human	Radiotherapy prognosis	Baek et al. (2020)
miR-218-5p↑	Serum	Human	—	Chen et al. (2021)
miR-29a-3p↓, miR-150-5p↓	Plasma	Human	IR-induced organ injury	Dinh et al. (2016)
miR-92a-1-5p↓, miR-25-5p↓, miR-1290↑	Serum	Human	Radiotherapy prognosis	Fan et al. (2020)
miR-34a↑	Serum	Human	Dose estimation	Halimi et al. (2016)
miR-26b-5p↓	Serum	Human	Radiotherapy prognosis	Han et al. (2020)
Combination of 16 miRNAs(e.g. miR-100-5p, miR-106b-5p, miR-145-5p)	Serum	Human	IR-induced organ injury	Hawkins et al. (2019)
miR-143↓	Serum	Human	Radiotherapy prognosis	Hiyoshi et al. (2017)
miR-1281↓, miR-6732-3p↓	Serum	Human	Radiosensitivity	Li et al. (2020a)
miR-6731-5p↑, miR-208b-3p↑, miR-2116-3p↓, miR-574-3p↓etc.	Exosomes in plasma	Human	Radiotherapy prognosis	Li et al. (2020b)
miR-130a↑, miR-25↑, miR-191*↑	Serum	Human, mouse	Radiotherapy prognosis	Lv et al. (2020)
Combination of miR-425-5p, miR-21-5p, miR-106b-5p, miR-590-5p, miR-574-3p, miR-885-3p	Plasma	Human	Dose estimation	Summerer et al. (2013)
miR-142-3p, miR-186-5p, miR-195-5p, miR-374b-5p and miR-574-3p	Plasma	Human	Radiotherapy prognosis	Summerer et al. (2015)
Combination of 11 miRNAs(e.g. miR-10b-5p, miR-125b-5p, miR-126-3p)	Serum	Human	Radiosensitivity	Sun et al. (2018)
miR-208a↑, miR-200a-3p↓, miR-126-3p↓, miR-29b-3p↓	Serum	Human	—	Tang et al. (2016)
Combination of miR-425-5p, miR-185-5p	Serum	Human	IR-induced organ injury	Tomasik et al. (2021)
Combination of miR-155, miR-221	Serum	Human	IR-induced organ injury	Xu et al. (2014)
miR-345↓	Serum	Human	Radiotherapy prognosis	Yu et al. (2016)
miR-504↑	Serum	Human	Radiotherapy prognosis	Zhao et al. (2015)

Researchers have been working to develop good radiation responsive molecules as radiation biodosimeters and some progress has been made on the use of miRNA expression in serum of animal models to assess the exposure dose. Jacob et al. (Jacob et al., 2013) used the Nano String nCounter system to compare miRNA expression in the serum of control and irradiated mice after 24 h of total body irradiation with 1, 2, 4, 6 and 8 Gy γ-rays. The system is based on miRNA fluorescence counting and the investigators found that the expression of miR-150, miR-200b and miR-762 was dose-dependent. The expression of miR-150 decreased significantly as the dose increased. miR-200b and miR-762 were the molecules whose expression increased in serum after irradiation, with more increases at higher doses (6 and 8 Gy). Due to the great dose effect of miR-150, the investigators further analyzed its downregulation at 24 and 48 h after irradiation. Compared to the control group, serum miR-150 expression levels decreased by 30% in the 1 Gy group after 24 h of TBI and continued to drop to 50% after 48 h. The time-and dose-dependent decline in miR-150 confirmed the sensitivity and stability of serum miRNA as a radiation biodosimeter. In a newly published study by that team, investigators delved into the achievability of miR-150-5p as a radioactive serum marker (Yadav et al., 2020). They determined the main source of circulating miR-150-5p was T and B lymphocytes (which are among the most sensitive cells to radiation) by miRNA expression profiling of sorted blood cells. This study confirmed miR-23a-3p which was abundant in blood was not radiation-responsive, and thus the investigators selected miR-23a-3p as an internal reference molecule for normalization and then used the ratio of miR-150-5p to miR-23a-3p expression (miR-150-5p/miR-23a-3p value, miR-RAD) for analysis. Firstly, serum was collected before and after fractionated irradiation [12 Gy (6 × 2 Gy) fractionated irradiation] in leukaemia patients, and they found a significant decrease of miR-RAD after irradiation with the expression gradually returning to baseline levels when patients received stem cell transplantation, suggesting the miR-RAD sensitivity for dose-response analysis. Subsequently, mouse models were conducted with exposure to gamma radiation at doses of 0.5, 1, 1.5, 2, 2.5, 3, 3.5, 4, 6, 8 and 10 Gy. A similar trend of radiation dose-response between the decline of miR-RAD and lymphocyte depletion was observed in mice and researcher confirmed their strong correlation. Radiobiological studies showed that total body exposure ≥2 Gy resulted in victims’ reduced lymphocyte counts and immune suppression (Singh et al., 2015). Therefore, the accepted clinical triage threshold is a 2 Gy dose at 24 h post-irradiation (DiCarlo et al., 2011). This study has shown that miR-RAD can differentiate between exposure to 2 Gy and controls in mice at 6–168 h post-radiation exposure. It may be particularly useful for screening victims with radiation exposure in the range of 1–3 Gy. Bugden et al. (Bugden et al., 2019) identified that global miRNA expression in the plasma of old mice was significantly decreased from that of young mice, and low dose radiation (10 mGy or 100 mGy) induced up-regulation of some low-abundance miRNA expression in old mice, which means low dose radiation might change the effect of aging on the global miRNA expression. They also found several miRNAs(up-regulated: miR-329-3p, miR-1249-3p, miR-6366, miR-703, miR-344g-5p; down-regulated: let-7j, miR-20a-5p) could be potential biomarkers for low dose radiation exposures. For high dose of radiation, Zhang et al. (Zhang et al., 2020) found that miR-151-3p and miR-128-3p could be used as dose-specific biomarkers of 8 Gy of IR exposure by detecting the expression of the 2 miRNAs in serum exosomes. Cui et al. (Cui et al., 2011) quantitatively evaluated the accuracy, sensitivity and specificity of the plasma miRNA biomarkers they found in mice, and they used a cluster analysis algorithm to identify the smallest set of miRNAs for predicting specific radiation doses at 6 and 24 h post-irradiation. The miRNA set at 6 h contained 32 miRNAs(miR-20b-5p, miR-190b, miR-339-3p, miR-466d-3p, miR-142-5p, miR-128a, miR-685, miR-339-5p, miR-532-5p, miR-378*, miR-690, miR-34a, miR-342-3p, miR-146a, miR-150, miR-361, miR-29c, miR-381, miR-155, miR-148a, miR-148b-5p, miR-652, etc.), and the 24h miRNA set contained 12 miRNAs (miR-764-5p, miR-205, miR-34a, miR-690, miR-302a, miR-361, miR-150, miR-34c*, miR-694, miR-463, miR-678 and miR-146b). The overall accuracy of both datasets was above 90%, and the specificity as well as the sensitivity were both above 95%. These miRNA signatures that were able to distinguish mice receiving 0.5, 2 or 10 Gy (6-or 24-h post-exposure) further supported why researchers regarded miRNA as a potential biomarker for radiation injury. In addition, a number of studies about miRNA biomarkers using animal models are also summarized in Table 2.

**TABLE 2:** Serum or plasma miRNAs as IR biomarkers in animal models.

miRNAs	Material	Species	Predictors	References
miR-130a-3p↑, miR-142-5p↓, miR-17-3p↓, miR-194-5p↑etc.	Serum	Mouse	IR-induced organ injury	Acharya et al. (2015)
↑: miR-329-3p,miR-1249-3p,miR-6366,miR-703,miR-344g-5p	Plasma	Mouse	Dose estimation	Bugden et al. (2019)
↓: let-7j, miR-20a-5p				
↑: miR-714, miR-3971, miR-150-5p, miR-5120, miR-5104	Serum	Mouse	—	Chakraborty et al. (2020)
↓: miR-204-5p, miR-96-5p, miR-124-3p, miR-9-5p, miR-211-5p				
miR-375-3p↑, miR-709↑	Serum	Mouse	IR-induced organ injury	Chiba et al. (2018)
miR34a↑, miR-142-5p↑etc.	Plasma	Mouse	Dose estimation	Cui et al. (2011)
miR-133b↑, miR-215↓, miR-375↑	Serum	Non-human primates	IR-induced organ injury	Fendler et al. (2017)
miR144-5p↑, miR-144-3p↑, miR-142-5p↑, miR-19a-3p↑	Whole blood	Rat	—	Gao et al. (2017)
miR-690↑, miR-223↑	Serum	Mouse	IR-induced organ injury	Islam et al. (2015)
miR-150↓, miR-200b↑, miR-762↑	Serum	Mouse	Dose estimation	Jacob et al. (2013)
miR-34a↑	Serum	Mouse	Dose estimation	Liu et al. (2011)
miR-150-5p↓, miR-574-5p↑, miR126↑, miR-144↑, miR-21↑, miR-1-3p↑, miR-206↑	Plasma	Non-human primates	Dose estimation	Menon et al. (2016)
Total miRNAs	Plasma	Mouse	Radiotherapy prognosis	Minnier et al. (2021)
miR-34a-5p, miR-34b-3p, miR-96-5p, miR-100-5p, miR-122-5p, miR-150-5p, miR-199a-5p, miR-199b-5p and miR-802-5p	Plasma	Mouse	Radiotherapy prognosis	Rogers et al. (2020)
miR-199a-3p, miR-25-3petc.	Plasma	Non-human primates	Radiotherapy prognosis	Rogers et al. (2021)
Combination of miR-183-5p, miR-9-3p, miR-200b-5p, miR-342-3p, miR-574-5p	Serum	Mouse	—	Wei et al. (2017)
miR-150-5p↓	Whole blood, serum	Mouse, human	Dose estimation	Yadav et al. (2020)
miR-101a↓, miR-103↓, miR-3096-5p↓, miR-652*↓	Serum	Mouse	IR-induced organ injury	Yamaguchi et al. (2019)
miR-151-3p↑, miR-128-3p↑	Exosomes in serum	Mouse	Dose estimation	Zhang et al. (2020)

Existing biological dose estimation techniques cannot effectively predict the severity of radiation injury, and triage is difficult to distinguish casualties with different levels of radiation injury, thus affecting subsequent medical treatment. In addition to the optimization of radiation biologic dose estimation techniques, research on radiation biomarkers has gradually advanced to find markers to assess radiation injury. The main symptoms of ARS are hematopoietic injury and gastrointestinal injury. Additionally, the injury caused by radiotherapy toxicity in cancer patients includes radiation lung injury, brain injury, etc. miRNAs have been reported to be involved in IR-induced organ injury, and exosome-derived miRNAs can be used to mitigate radiation injury. Rogers et al. demonstrated that early expression levels of some plasma miRNAs can be used to predict radiation-induced pneumonia and pulmonary fibrosis in mice (Rogers et al., 2020) and non-human primates (Rogers et al., 2021). Through KEGG pathway analysis they identified the role of miR-34a-5p, miR-100-5p and miR-150-5p in NF-κB-driven acute pro-inflammatory pathway, and the involvement of miR-34b-3p, miR-96-5p and miR-802-5p in fibrogenic TGF-β-SMAD signaling pathway. Liu et al. (Liu et al., 2019) found that miR-22 overexpression in bone marrow mesenchymal stem cells irradiated with 6Gy dose (obtained from SD rat femurs) accelerated IR-induced accumulation of reactive oxygen species in mitochondria, and attenuated IR-activated cellular autophagy, thus promoting apoptosis, which showed reduced viability of bone marrow mesenchymal stem cells and suppression of osteogenic capacity at the organ level. Lei et al. (Lei et al., 2021) found that miR-214-3p delivered to mice via MSC exosomes could inhibit the ATM/P53/P21 signaling pathway to mitigate IR-induced lung injury. Leavitt et al. (Leavitt et al., 2020) found that human neural stem cell-derived exosomes could alleviate IR-induced cognitive dysfunction in mice through a mechanism involving miR-124. miR-378a-3p was reported to protect the small intestine from ionizing radiation toxicity by inhibiting CDK6 expression (Chu et al., 2020). miR-122-5p was found to aggravate IR-induced rectal injury by targeting CCAR1 and *in vivo* injection of miR-122-5p antagomir after irradiation significantly mitigated IR-induced rectal injury in mice. Muhammad et al. (Muhammad et al., 2021) applied Surface-enhanced Raman spectroscopy (SERS) to detect miR-122 expression in serum exosomes after radiation, and this technique has high sensitivity and specificity for assessing radiation-induced liver injury. In a word, it is clear that some miRNAs have been shown to be involved in the biological response to IR damage in an organ and can be used as predictors of radiation injury, however, the detection of these miRNAs’ expression in serum or plasma is less reported and further validation is needed.

Serum miRNA biomarkers that predict radiation injury in research reflect the degree of radiation injury in the body through the survival of the individual after clinical treatment. One study (Acharya et al., 2015) found that a group of five miRNAs (miR-130a-3p, miR-142-5p, miR-150-5p, miR-706 and miR-342-3p) were able to distinguish between groups of mice irradiated with 0 or 2 Gy at 24 h after TBI of gamma rays, and another group of three miRNAs(miR-34b-3p, miR-126-3p and miR-17-3p) were able to distinguish between groups irradiated with 2 and 6.5 Gy (representing low and high sublethal doses respectively). A third group of five miRNAs (miR-187-3p, miR-194-5p, miR-30c-5p, miR-27a-3p and miR-30a-3p) was able to distinguish between groups irradiated with 6.5 and 8 Gy (representing sublethal and lethal doses, respectively) at 24 h. Notably, none of the miRNAs in these groups were able to discriminate between 0 and 2 Gy, 2 and 6.5 Gy, or 6.5 and 8 Gy at the same time, possibly because the aim of this study was to identify the set of miRNAs with the greatest differences at different radiation doses. Therefore, there may be other miRNAs that are able to distinguish between a larger range of radiation doses. These sets of miRNAs found the relevance of serum miRNAs to the effects of radiation, and in particular the discovery that serum miRNAs that can distinguish between sublethal (6.5 Gy) and lethal (8 Gy) exposures may be associated with hematopoietic damage and animal viability. This is crucial because in a radiation accident emergency, immediate determination of whether a person has been exposed to lethal ionizing radiation is more important than dose estimation in early medical treatment. Afterwards, this study elucidated the correlation between miRNAs and the degree of radiation damage. They used the radioprotective agent amifostine, which is known to prolong survival in mice and humans by reducing radiation-related toxicity (Pamujula et al., 2005; Molkentine et al., 2019; Singh and Seed, 2019), to inject mice intraperitoneally prior to irradiation. The results showed that all mice in the amifostine injection group (before IR) survived, and all the mentioned miRNAs above were significantly altered compared to the saline injection group (before IR), suggesting that this group of miRNAs was related to the ability to survive (or the degree of damage suffered) and not the radiation dose. Similarly, bone marrow transplantation after irradiation also provided a protective effect in terms of prolonged survival, and this group of miRNAs (miR-187-3p, miR-27a-3p, miR-30a-3p and miR-30c-5p) also predicted the survival of bone marrow MSC-transplanted animals after irradiation. The above revealed that serum miRNA profiles can not only serve as biological dosimeters, but can indicate the degree of damage caused by radiation and the survival. To demonstrate that post-irradiation serum miRNAs can be used as predictive markers of mortality following irradiation in humans, the team used non-human primates to testify the conservation of these miRNAs across species in 2017 (Fendler et al., 2017). They found a combination of three miRNA radiation biomarkers (miR-133b, miR-215 and miR-375) to distinguish potential irradiated subjects, indicators used to predict survival (miR-30a and miR-126) and some miRNAs(miR-142 and miR-320a) for normalization. And then researchers combined them into an algorithm model that could successfully distinguish whether people has been exposed to radiation and accurately estimate the probability of death within 24 h of IR exposure.

### Urine

Bhayana et al. (2017) identified that miR-1224 and miR-21 in urine could be used as early and late radiation-responsive markers of radiation nephropathy, respectively. Their study concluded that early-responding miRNAs probably reflect the degree of injury in a tissue via miRNAs’ counts, whereas the late-responding miRNAs are likely to show cascade of responsive pathways that are activated as a function of radiation exposure. A recent study (Zhou et al., 2021) found a significant difference of miR-223-3p expression between the urine of radiation-resistant and radiation-sensitive prostate cancer patients. When established miR-223-3p overexpression and knockdown cell models, researchers found that this molecule affected the radiosensitivity of prostate cancer cells. It is reported that urinary miRNAs could be disease biomarkers for urological cancers such as prostate, bladder and renal cell cancers (von Brandenstein et al., 2012; Blanca et al., 2017; Fredsøe et al., 2018; Jeon et al., 2020), and currently miRNA has been reported to modulate radiosensitivity, while there are few studies on the alteration of miRNAs in urine after radiation therapy. Exploring the alteration of miRNAs in urine after irradiation could promote the development of auxiliary indicators to monitor the prognosis of radiotherapy in the future.

### Other Body Fluids

In addition to blood and urine, salivary miRNAs were reported in association with IR. A study have been carried out in HNSCC patients (Ahmad et al., 2020). The study identified some miRNAs in the saliva of patients who received intensity modulated radiation therapy (IMRT) by RNA sequencing. They detected those miRNAs by Real-time quantitative PCR(RT-qPCR) to assess whether salivary miRNAs are the potential predictors for IMRT in patients with squamous cell carcinoma of the head and neck. Finally they found that salivary miR-15a-5p was associated with progression-free survival after treatment and then speculated that it might be an independent predictor of local progression-free survival. Tear miRNAs have also been reported to be used as a disease biomarker for tumors (Inubushi et al., 2020), researchers found breast cancer-specific miR-21 and miR-200c were highly expressed in tear exosomes from patients with metastatic breast cancer compared to those from healthy volunteers.

The accessibility and stability of miRNAs in body fluids and the application of miRNAs as disease biomarkers suggest the promise of miRNAs in body fluids (e.g., serum) as markers of IR. Some studies have reported the use of a combination of several miRNAs as indicators to differentiate between different IR exposure doses (Fendler et al., 2017). This is because the alteration of a single miRNA expression may be unstable due to individual differences or other environmental factors, so using a combination of several miRNAs as indicators will provide more convincing results and balance specificity and accuracy. Accurate quantitative detection of miRNAs is the basis for their use as biomarker, RT-qPCR, Northern blotting, Next generation sequencing and Microarray analysis have been described for miRNA quantification (Bartels and Tsongalis, 2009). RT-qPCR has become the gold standard for measuring miRNA expression due to its high sensitivity, specificity, reproducibility and low template requirements (Schmittgen and Livak, 2008). Absolute quantitative assays are not optimal for accurate detection of miRNA expression levels because of the high quality of the RNA required. Similar to gene expression analysis, relative quantification is the preferred method for miRNA expression analysis. However, there is no standard reference molecules, and thus we need to find the best standardization method for cell-free miRNA data. Currently there are exogenous and endogenous reference molecules, and many researchers choose to use the introduction of synthetic small RNA *in vitro* [e.g., miR-39 from *C. elegans* (Murray et al., 2016; Franck et al., 2020; Parker et al., 2021) and miR-54 (Kuhlmann et al., 2014) etc.] as reference controls. Many internal reference genes, such as small nuclear/nucleolar RNAs, have been commonly used to quantify miRNA expression (Zhu et al., 2012; Serafin et al., 2014; Li et al., 2015). However, their biosynthesis and tissue-specific expression are not similar with miRNAs, and thus those small RNAs are less suitable for normalization of miRNA expression theoretically. miR-16 has been reported as a stably expressed miRNA in multiple samples, but it is not consistently expressed under various external treatment conditions, for instance miR-16 expression was affected by IR (Bersimbaev et al., 2020). Hence, different molecules for normalization should first be established for different samples, and combinations of several molecules may be more appropriate than a single generic molecule.

## Exploration of the Source and Destination of miRNAs in Serum After Ionizing Radiation

While exploring the potential of miRNAs in serum as biomarkers of ionizing radiation damage, the sources and destinations of some miRNAs under ionizing radiation condition are unknown. Various cells can release miRNAs into other tissues, and these miRNAs act as a form of intercellular signaling to alter the function of receptor cells by regulating the expression of target genes (Zhang et al., 2010; Chen et al., 2012). So which organs do miRNAs come from after exposure to IR and where do they go through the circulation or other body fluids? There are some articles that have done pre-exploration in this area, and they are summarized below.

### Possible Sources of miRNAs After Ionizing Radiation

In addition to confirming that some miRNAs (miR-425-5p, miR-93-5p) in the plasma of HNSCC patients can be used as markers for radiotherapy in HNSCC, Summerer et al. (Summerer et al., 2013) also tried to find the cellular origin of these differentially expressed miRNAs, and they hypothesized that plasma differentially expressed miRNAs after radiotherapy originate from PBMCs in the same patients, based on the theory that many known plasma miRNAs originate from PBMCs (Hunter et al., 2008) as reported in the article. Subsequent experimental results showed no significant correlation between PBMCs and plasma miRNA expression in patients before and after treatment, leading to the conclusion that these plasma miRNAs may not have originated from PBMCs in the blood. As previously reported, some researchers hypothesized that plasma miRNAs were derived from tumor cells, either actively secreted by tumor cells or present in apoptotic vesicles released from dead tumor cells. So they speculated that miRNAs may originate from tumor cells after IR, and subsequently cultured primary HNSCC cells *in vitro* under conditions that mimic radiotherapy *in vivo*. The results revealed that miR-425-5p, miR-21-5p, miR-106b-5p and miR-93-5p were differentially expressed in HNSCC cells cultured *in vitro*. miR-425-5p was down-regulated in plasma and up-regulated in cells, and miR-93-5p was up-regulated in plasma and down-regulated in cells. Based on the expression of miR-93-5p in two models, the researchers thus concluded that miRNAs were released by tumor cells following injury. In their follow-up study, a proportion of radiotherapy-responsive miRNAs (miR-21-5p, miR-28-3p, miR-142-3p, miR-191-5p, miR-186-5p, miR-197-3p, miR-425-5p) were also tumor-specific miRNAs, and all of them were detected in the local tumor tissues of patients, indicating that these miRNAs were associated with tumors. However, the expression of miRNAs in tumors did not significantly correlate with the one in patients’ plasma, which may be related to the fact that the partial tumor tissues detected were not representative of the overall tissue expression.

Chiba et al. (2018) used X-rays to induce acute radiation syndrome in mice by TBI at 7 Gy and found that miR-375-3p and miR-709 expression were significantly elevated in serum and could be candidate serum biomarkers for acute radiation syndrome. The investigators also explored the origin of the two miRNAs. The expression of miR-375-3p and miR-709 in normal mouse tissues and organs was first examined. Total RNA was extracted and quantified respectively from peripheral blood leukocytes and bone marrow cells and 18 organs (brain, eye, salivary gland, thymus, heart, liver, stomach, pancreas, kidney, spleen, lung, testis, prostate, bladder, seminal vesicles, intestine, colon and muscle). The results showed that miR-375-3p and miR-709 were highly expressed in the pancreas, so researchers speculated that radiation-induced high abundant miR-375-3p and miR-709 in serum may be released from the pancreas. The expression of the two miRNAs in the pancreas after 7 Gy X-ray TBI was then investigated and results showed that miR-375-3p expression was down-regulated in pancreatic tissue 48 and 72 h (0.68-fold and 0.41-fold, respectively) after 7 Gy irradiation compared to the control group. miR-709 expression was not significantly different in the pancreas. As miR-375-3p expression was also relatively high in the intestine, they also analyzed miR-375-3p expression in the small intestine of two groups. miR-375-3p was down-regulated in the intestine at 24, 48 and 72 h post-irradiation (0.69-fold, 0.83- fold and 0.62-fold, respectively), suggesting that radiation-induced cell death may result in miR-375-3p being released from cells into the extracellular space and then lead to its high expression in serum.

Two mechanisms have been proposed for the source of reduced miRNAs in serum or plasma following radiation: reduced cellular exosome secretion or reduced miRNAs entry into exosomes. Exosomes, a variety of vesicular structures with a membrane structure released by cells, are present in all body fluids (Simons and Raposo, 2009; Mathivanan et al., 2010), exosome’s contents contain proteins, lipids and RNA [including non-coding RNAs such as miRNAs (Sato-Kuwabara et al., 2015)] and are involved in intercellular biological signaling. Exosomes have been described as an integral part of the cellular stress response, including radiation exposure (Mutschelknaus et al., 2016). Dinh et al. (Dinh et al., 2016) found that plasma miR-29a and miR-150 decreased with increasing irradiation dose in patients with non-small cell lung cancer after radiotherapy. Subsequent detection of corresponding miRNA expression in exosomes from *in vitro* cultured non-small cell lung cancer cells showed that miR-29a and miR-150 expression were significantly reduced in exosomes secreted from cells into the culture medium after irradiation, whereas miRNA expression increased in non-small cell lung cancer cells after irradiation. This suggests that the decrease in miR-29a and miR-150 levels might be a regulated process. The author hypothesized that both tumor and non-tumor cells may reduce miRNA transportation from intracellular to extracellular by reducing exosome secretion or by reducing miRNAs loading into exosomes, resulting in an accumulation of intracellular miRNAs. Both mechanisms are radiation effects worthy of further investigation and provide important implications for the role of miRNA involvement in mitigating radiation-induced damage.

According to the results of the above studies, it could be speculated that a proportion of miRNAs in serum or plasma are secreted from the tissues or organs where they are abundant (normal or tumorous tissues), and exposure to IR damages the corresponding tissues or organs, thus affecting the secretion of miRNAs into the circulation and resulting in abnormal serum or plasma miRNA expression. Although it can be demonstrated in mouse models that the high expression of miRNAs in serum or plasma is derived from damaged cells, the results can not be repeated in human. Two hypotheses regarding the source of miRNAs with reduced expression in serum or plasma that need to be further proved by more experimental evidence.

### Possible Destinations of miRNAs After Ionizing Radiation

Given the signature of tissue-specific miRNAs, changes in miRNA expression levels in different parts of the body following exposure to IR may suggest the destination of miRNAs.

miRNA’s hormone-like effects have been reported probing what function the miRNAs secreted into receptor cells exert (Bayraktar et al., 2017). Zhang et al. (Zhang et al., 2018) found that pancreatic islet cells secrete miRNAs via exosomes, which can be transported to receptor tissue cells and regulate gene functions. In this study, the researchers demonstrated that under physiological conditions islet cells could selectively secrete miRNAs and store them all in exosomes. They found that miR-223 secreted by pancreatic islet cells promoted the expression of GLUT4 in those tissues, such as skeletal muscle and liver tissue in obese mice, thereby improving glucose uptake. Thus, tissues and organs in the body can secrete miRNAs to other tissues for regulation under physiological and pathological conditions.

According to the above research, some miRNAs may play a similar role in radiation conditions. Actually, an article reported their findings about the speculation. Moertl et al. (Moertl et al., 2020) found a dose-dependent expression of miRNAs in radiation-induced release of extracellular vesicles from PBMCs, and further they explored the alterations in receptor cells affected by miRNAs from PBMCs. The researchers co-cultured fluorescently labelled extracellular vesicles with PBMCs and endothelial cells *in vitro* and observed the uptake of extracellular vesicles by both types of cells when unirradiated and irradiated. The results showed that irradiation induced enhanced uptake of extracellular vesicles by endothelial cells. The researchers then investigated whether PBMC-secreted extracellular vesicles were taken up by endothelial cells and whether the substances in extracellular vesicles inhibited endothelial cell apoptosis. Isolated extracellular vesicles from unirradiated or irradiated (0, 0.1, 2, and 6 Gy) PBMCs were co-incubated with endothelial cells, and results showed low levels of apoptosis after irradiation of endothelial cells, suggesting that these extracellular vesicles have anti-apoptotic properties. miR-23a and miR-101-3p may contribute to the inhibition of endothelial receptor cell apoptosis (Heider et al., 2017; Kim et al., 2017). Moertl et al.’s study suggests that endothelial cells receive extracellular vesicles from PBMCs after radiation, and inhibit radiation-induced apoptosis.

Elucidating the source and destination of serum miRNAs after IR could explain what roles miRNAs play in organism. Most of the studies have validated miRNA release from donor cells and uptake by receptor cells *in vitro*, but studies based on physical circulation are few. Research whether miRNAs passively or actively participate *in vivo* after exposure to IR is worthy of further investigation.

## Conclusion

Although a variety of biomarkers have been considered for dose estimation and damage assessment after radiation exposure, the development of new ionizing radiation marker molecules could help to further enrich the types of indicators for effective clinical triage. MiRNAs have been extensively studied as disease biomarkers due to their stable presence in body fluids, and current research in radiobiology has progressively elucidated the involvement of miRNAs in regulating the biological effects induced by IR. Moreover, researchers also identified several time-specific and dose-specific miRNAs and some miRNAs for radiation injury assessment in animals and patients’ samples. While challenges still remain in using miRNAs as radiation biodosimeters and indicators of radiation injury, the issues that researchers need to further solve are: (i) We need to find more sensitive radiation-specific miRNAs. (ii) In addition to the need for high sensitivity, specific timepoints and doses after IR exposure are similarly important. The expression of miRNA is dynamically changing, and it is necessary to identify appropriate miRNAs at specific timepoints or doses according to the purpose of the clinical examination. (iii) It is essential to take individual differences into account. (iv) Suitable clinical samples need to be selected for validation. What’s more, explaining the source of miRNAs in body fluids and miRNA signals in receptor cells or tissues after exposure to IR would also be the focus of subsequent research. That would help to understand the role of miRNA in radiation response at the organ level, as well as consider the possibility of miRNA applications in radiation protection and clinical treatment.
